# Targeting neuroimmune interactions: the therapeutic potential of kaempferol in immune-related central nervous system disorders

**DOI:** 10.3389/fimmu.2026.1837418

**Published:** 2026-07-15

**Authors:** Yanan Zou, Jianting Huang, Junbo Yin, Lei Liu, Haipeng Zhou, Baizhou Song, Yifei Wang, Mengyan Wang, Lezhen Wang, Xiangyi Kong, Fei Rong

**Affiliations:** 1 Qingdao Mental Health Center, Qingdao, China; 2 Department of Anesthesiology, Qilu Hospital (Qingdao), Cheeloo College of Medicine, Shandong University, Qingdao, China

**Keywords:** blood brain barrier, kaempferol, neuroinflammation, neurological disorders, reactive oxygen species

## Abstract

Neuroinflammation is recognized as a pivotal pathological process underlying a spectrum of neurological disorders. The exploration of natural flavonoids as therapeutic agents has substantially advanced our understanding of strategies to mitigate neuroinflammatory injury. Accumulating evidence indicates that kaempferol—a dietary flavonoid abundantly present in various fruits and vegetables—exerts potent neuroprotective effects in multiple neurological conditions. Its beneficial actions are mediated through multi-target mechanisms, primarily involving the suppression of microglial activation, modulation of immune cell reactivity, and enhancement of endogenous antioxidant defenses. These mechanisms collectively contribute to reduced production of inflammatory mediators, alleviation of oxidative stress, and inhibition of neuronal apoptosis, thereby counteracting the pathogenesis of various neuroinflammatory diseases. This review summarizes current knowledge on the protective role of kaempferol in the pathogenesis and progression of central nervous system disorders. We further elucidate the underlying molecular and cellular mechanisms, as well as autophagy and oxidative stress. Additionally, potential challenges in clinical translation, such as bioavailability and blood-brain barrier permeability, are discussed to guide future research in this promising field. Elucidating the pleiotropic actions of kaempferol will not only deepen our understanding of its pharmacodynamics but may also open new avenues for the prevention and treatment of neuroinflammatory-related neurological diseases.

## 1 Introduction

Neuroinflammation, a specialized inflammatory response within the central nervous system (CNS), plays a pivotal role in the pathogenesis of various neurological disorders, including Alzheimer’s disease (AD) (1), Parkinson’s disease (PD) (2), multiple sclerosis (MS) (3), and stroke (4). Distinct from peripheral inflammation, neuroinflammation is characterized by the activation of CNS-resident immune cells, most notably microglia and astrocytes. In response to sustained stimulation by various pathological triggers including abnormal protein aggregates and damage-associated molecular patterns (DAMPs) these glial cells secrete elevated levels of pro-inflammatory cytokines, chemokines, and reactive oxygen species (ROS). This persistent inflammatory milieu contributes directly to neuronal injury and impairs synaptic plasticity, thereby accelerating disease progression (5). While acute neuroinflammation is crucial for tissue repair and pathogen clearance, chronic neuroinflammation drives progressive neurodegeneration and cognitive decline (6). This transition from a protective to a detrimental state represents a core event in neurodegenerative diseases. Given the limitations of conventional anti-inflammatory drugs—such as non-steroidal anti-inflammatory drugs and corticosteroids, which often exhibit poor blood-brain barrier (BBB) penetration and significant side effects with long-term use—there is growing interest in identifying natural compounds with effective anti-inflammatory properties and more favorable safety profiles.

Kaempferol (3,4′,5,7-tetrahydroxyflavone) is a natural flavanol widely present in common dietary sources and medicinal plants, notably in green vegetables, various fruits, and herbal medicines. By virtue of its unique phenolic hydroxyl structure, kaempferol exhibits significant therapeutic potential, demonstrating potent antioxidant (7), anti-inflammatory (8), and anticancer (9) activities. Preclinical evidence indicates that kaempferol confers neuroprotection via multifaceted mechanisms, such as attenuating microglial activation, downregulating Nuclear Factor-kappa B (NF-κB) and NLR Family Pyrin Domain Containing 3 (NLRP3) inflammasome pathways, and decreasing the expression of pro-inflammatory cytokines (8). For instance, in lipopolysaccharide (LPS)-induced neuroinflammation models, kaempferol was found to inhibit the production of tumor necrosis factor-α (TNF-α), interleukin-6 (IL-6), and IL-1β (10). In atherosclerosis research, kaempferol enhanced antioxidant effects via activation of the Nuclear factor erythroid 2-related factor 2 (Nrf2)/heme oxygenase-1 (HO-1) pathway (11). Furthermore, kaempferol can cross the BBB, thereby augmenting its protective role. It has been shown to mitigate LPS-induced neuroinflammation and BBB dysfunction by inhibiting HMGB1 release and downregulating the TLR4/MyD88 pathway (12). This multi-target capacity, simultaneously addressing inflammation, oxidative stress, and barrier integrity, positions kaempferol as a promising dietary supplement for the prevention and treatment of neurodegenerative diseases (13).

The multi-target efficacy of kaempferol has been specifically validated across different disease models. In AD animal models, kaempferol reduced the deposition of β-amyloid (Aβ) plaques and tau hyperphosphorylation while improving cognitive function (14), effects closely associated with its inhibition of Aβ-induced microglial activation. Similarly, in PD models, kaempferol protected dopaminergic neurons by inhibiting α-synuclein aggregation and microglia-mediated neurotoxicity (8). These findings collectively suggest that kaempferol can exert therapeutic benefits across a spectrum of neuroinflammation-related conditions. However, the current body of evidence remains somewhat fragmented. A systematic discussion and comparison of kaempferol’s anti-inflammatory effects across different neurological diseases is lacking, and the complete map of its specific signaling network has yet to be fully elucidated.

Therefore, this review seeks to systematically synthesize current evidence and offer a comprehensive overview of the mechanistic actions of kaempferol in the context of neuroinflammation. It will critically evaluate its therapeutic potential in specific neuroinflammation-associated diseases such as AD, PD, and stroke. Additionally, it will delve into strategies to enhance its translational applicability—including addressing its bioavailability and exploring formulation improvements—to provide a clear direction for future research and clinical development of this natural compound.

## 2 Synthesis and metabolism of kaempferol

### 2.1 Chemical properties of kaempferol

Kaempferol (3,5,7-trihydroxy-2-(4-hydroxyphenyl)-4H-1-benzopyran-4-one), also referred to as triflorin or kaempferol, is a naturally occurring flavonoid belonging to the flavonol subclass. Its molecular formula is C_15_H_106_, corresponding to a molecular weight of 286.23 g/mol. The pure compound typically presents as a yellow crystalline powder with a melting point range of 276–278 °C (15). It exhibits low solubility in water but is soluble in hot ethanol, diethyl ether, dimethyl sulfoxide, and other organic solvents. In phytochemical research, flavonoids are commonly found as glycosides and are widely distributed across various plant species. Among them, kaempferol derivatives serve as important representatives of flavonols, exhibiting remarkable structural diversity in their glycosylated forms and demonstrating significant biological activities. Commonly reported kaempferol glycosides with well-documented bioactivities include astragalin (kaempferol-3-O-glucoside), populnin (kaempferol-7-O-glucoside), nicotiflorin (kaempferol-3-O-rhamnoside), and kaempferitrin (kaempferol-3,7-di-O-rhamnoside). These O-glycosides not only possess intrinsic pharmacological activities but also function as precursor molecules for further acylation modifications (16). The chemical structure of kaempferol is characterized by a 15-carbon skeleton arranged into two aromatic rings (A and B), interconnected by a heterocyclic pyran ring (C). Hydroxyl groups are positioned at the 3, 5, 7, and 4’ carbon atoms. These hydroxyl moieties, particularly the catechol group on the B-ring and the free 3-OH group, are crucial for its redox properties and metal-chelating capabilities (15). The biosynthesis of kaempferol initiates with the condensation of 4-coumaroyl-CoA with three malonyl-CoA molecules, a reaction catalyzed by chalcone synthase, yielding naringenin chalcone as the primary product (17, 18). Subsequently, chalcone isomerase mediates the stereospecific cyclization of naringenin chalcone to form the flavanone naringenin. The biosynthetic pathway then proceeds through the hydroxylation of naringenin at the C3 position, a reaction catalyzed by flavanone 3-hydroxylase, to generate dihydrokaempferol (17, 18). The final step involves the introduction of a C2–C3 double bond in the dihydrokaempferol skeleton through the action of flavonol synthase, resulting in the formation of kaempferol (17). *In planta*, kaempferol frequently undergoes glycosylation at various hydroxyl positions, with common sugar moieties including xylose (19), rhamnose, glucose, galactose, and the disaccharide rutinose (comprising glucose and rhamnose connected by an α-glycosidic bond) (20). This glycosylation leads to the production of derivatives such as astragalin (kaempferol-3-O-glucoside) (17).

### 2.2 Sources of kaempferol

**Figure 1:**
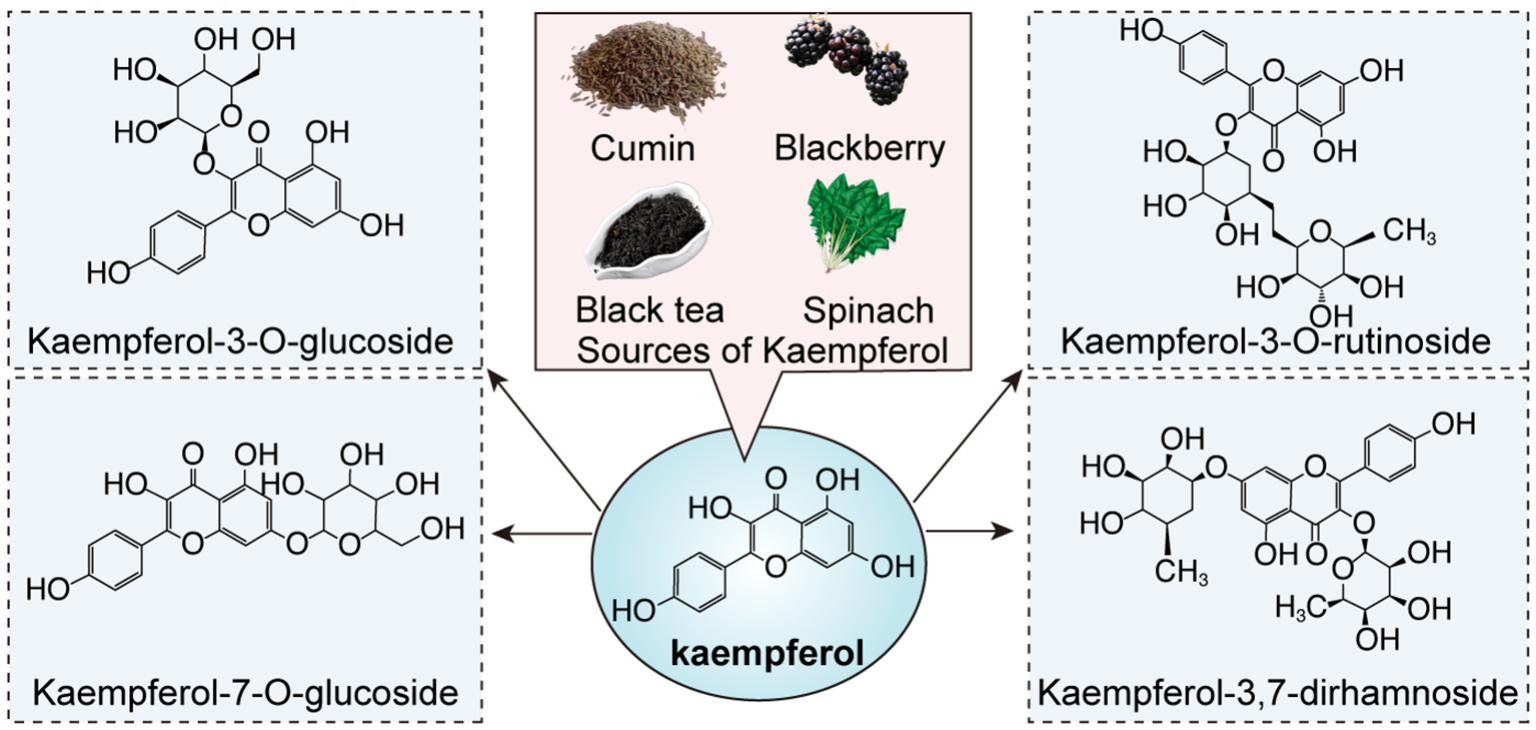
Chemical structures of kaempferol and its common glycosylated derivatives. Astragalin (kaempferol-3-O-glucoside), populnin (kaempferol-7-O-glucoside), nicotiflorin (kaempferol-3-O-rhamnoside), and kaempferitrin (kaempferol-3,7-di-O-rhamnoside).

The primary dietary sources of kaempferol include fruits, vegetables, tea, and certain medicinal plants such as *Ginkgo biloba* leaves and *Sophora flavescens* (13, 21, 22). Its distribution exhibits significant species and tissue specificity, with varying concentrations across different plants reflecting its diverse physiological and defense functions. In these plants, kaempferol predominantly exists in glycosylated forms. Common sugar moieties include glucose, rhamnose, and galactose. This glycosylation represents the primary form for its storage and transport, profoundly influencing its water solubility, cellular uptake efficiency, bioavailability, and ultimate bioactivity (23–26). Furthermore, kaempferol has been isolated from several plant families, including Berberidaceae, Fabaceae, and Asteraceae, underscoring its widespread occurrence in nature and its phylogenetic significance among plant secondary metabolites (27, 28). Recent studies have identified kaempferol in propolis and bee products, suggesting a role in plant-pollinator interactions and potential therapeutic applications (29, 30). This discovery extends the ecological function of kaempferol from the plant kingdom to the animal kingdom, implying potential indirect effects on human health through the food chain. The widespread distribution of this compound across various plant taxa underscores both its ecological significance and therapeutic promise, meriting continued exploration of its biosynthetic routes, regulatory networks, and full spectrum of health-related benefits (**Figure 1**).

### 2.3 Biometabolism of kaempferol

Kaempferol exhibits poor water solubility, undergoes extensive metabolism *in vivo*, and is rapidly eliminated, resulting in a relatively low oral bioavailability, which constitutes a major bottleneck limiting its clinical application (31, 32). Its *in vivo* journey begins in the intestine: ingested kaempferol glycosides are hydrolyzed by β-glucosidases within intestinal epithelial cells or hydrolases secreted by the gut microbiota, releasing the lipophilic aglycone for passive diffusion and absorption (33, 34). However, its absorption is constrained by active efflux mediated by transporters such as P-glycoprotein and multidrug resistance-associated proteins on the apical membrane of intestinal cells (5, 35, 36), forming the first barrier limiting its intestinal uptake. Once absorbed into enterocytes and the portal circulation, kaempferol undergoes extensive Phase II metabolism in the liver and intestinal epithelium, including glucuronidation, sulfation, and methylation, forming more polar conjugates (36–39). The major metabolites detected in plasma include kaempferol-3-O-glucuronide, kaempferol-7-O-sulfate, and isorhamnetin (3’-O-methylkaempferol). These conjugated derivatives not only exhibit bioactivities distinct from the aglycone but also determine its tissue distribution profile, being transported to various tissues and organs to exert therapeutic effects (40). Pharmacokinetic studies indicate that after intravenous administration, kaempferol distribution follows a one-compartment model, characterized by high clearance (4.40–6.44 L/h/kg) and a very short half-life (2.93–3.79 minutes). Its metabolites are primarily excreted via urine (renal) and bile (hepatic) (41). Notably, the gut microbiota plays a complex and crucial role in kaempferol metabolism, capable of not only converting its glycosylated precursors into the aglycone but also further degrading kaempferol into smaller phenolic acids, such as 4-hydroxyphenylacetic acid (4-HPAA). These microbial metabolites may possess unique bioactivities and could indirectly enhance the overall bioavailability and efficacy of kaempferol via pathways like the gut-brain axis (42–44).

**Figure 2:**
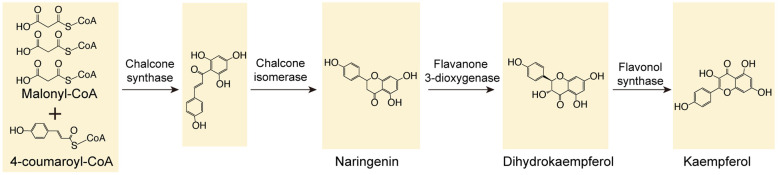
The biosynthetic pathway of kaempferol. Chalcone synthase catalyzes the condensation of one molecule of 4-coumaroyl-CoA with three molecules of malonyl-CoA to form naringenin chalcone. This intermediate is subsequently isomerized to naringenin by chalcone isomerase. The naringenin scaffold is then hydroxylated at the C3 position by arylketone-3 oxygenase, yielding dihydrokaempferol. Finally, flavanol synthase introduces a C2-C3 double bond into dihydrokaempferol to produce kaempferol.

Given its inherent pharmacokinetic limitations, researchers have explored various strategies to enhance its application value. Kaempferol has been reported to enhance the efficacy of other anticancer drugs through synergistic effects when used in combination; for instance, co-administration with quercetin significantly enhanced the anticancer affinity of the latter (45, 46). On the other hand, progress has been made in formulation improvements for kaempferol itself. Nanocapsulation to increase its solubility and stability, or structural derivatization to optimize its metabolic properties, have proven to be viable strategies for effectively improving the bioavailability and ultimate therapeutic efficacy of kaempferol (47, 48). Therefore, the current consensus suggests that, despite the limitation of low bioavailability when administered alone, kaempferol can exert greater value through two main pathways: first, acting as a sensitizer or synergist to enhance the therapeutic effects of various anticancer drugs; second, directly improving its own pharmacokinetic profile through advanced formulation engineering or prodrug design, thereby achieving superior therapeutic outcomes (**Figure 2**).

## 3 Kaempferol suppresses pro-inflammatory activation of immune cells in the central nervous system

### 3.1 Macrophages

The central nervous system contains a diverse population of resident macrophages, such as CNS-associated macrophages (CAMs), border-associated macrophages, and Kolmer’s epiplexus cells (also known as supraependymal macrophages). Among these, CAMs are strategically positioned within key neuroanatomical compartments—such as the brain parenchyma (cortical regions), meninges, choroid plexus, and perivascular spaces—where they continuously monitor the microenvironment. Emerging evidence indicates that CAMs play crucial regulatory roles in brain development, homeostasis, and neuroinflammatory diseases like MS and AD by modulating neuroimmune interactions, synaptic pruning, and vascular integrity (49–51). Notably, their dysregulation or maladaptive activation is increasingly recognized as a pivotal factor in the initiation and perpetuation of chronic neuroinflammation, rendering them important targets for therapeutic intervention. In this context, natural compounds with immunomodulatory properties offer promising avenues for intervention. Studies demonstrate that kaempferol exerts significant immunomodulatory effects on macrophages, influencing their polarization, inflammatory responses, and functional plasticity. Accumulating evidence indicates that kaempferol can suppress pro-inflammatory M1 macrophage activation while promoting anti-inflammatory M2 polarization, thereby contributing to inflammation resolution across various disease contexts (52, 53). The molecular mechanisms underlying these effects are multifaceted and have been elucidated in multiple experimental models. In THP-1-derived macrophages, kaempferol was found to inhibit IL-32-induced monocyte differentiation into macrophage-like cells and reduce the production of pro-inflammatory cytokines along with their mRNA expression. Specifically, kaempferol suppressed IL-32-induced activation of p38 and NF-κB, consequently ameliorating the production of inflammatory mediators—including TSLP, IL-1β, TNF-α, IL-8, and nitric oxide (NO)—in LPS-pre-stimulated, IL-32-differentiated macrophage-like cells (54). In the context of cardiovascular disease, kaempferol has been demonstrated to suppress foam cell formation through inhibition of the Piezo1 channel and subsequent calcium influx. This mechanism in turn modulates CD36-dependent mitochondrial ROS generation and influences downstream signaling cascades, including the NF-κB/Mitogen-Activated Protein Kinase (MAPK) and HO-1/Nrf2 pathways, underscoring its therapeutic potential against atherosclerosis (11). Consistent with this, studies in human THP-1 macrophages confirmed kaempferol’s anti-atherosclerotic and anti-inflammatory properties (55), attributed to its capacity to diminish key pro-inflammatory cytokines, promote the repolarization of microglia from the M1 to the M2 phenotype, and interrupt the ROS/NF-κB signaling axis (53).

The therapeutic scope of kaempferol extends beyond the CNS and cardiovascular systems. In a CLP-induced acute kidney injury model, kaempferol alleviated renal damage by modulating the infiltration of F4/80+ macrophages. Kaempferol supplementation has further been shown to ameliorate intestinal inflammation and enhance barrier function by suppressing the TLR4/NF-κB pathway. This leads to reduced infiltration of immune cells—including macrophages, dendritic cells, and neutrophils—and decreased expression of inflammatory mediators such as TNF-α, IL-1β, IL-6, and MCP-1. These effects collectively contribute to its therapeutic benefits in obesity management (56). Interestingly, in the pathogenesis of obesity-induced chronic inflammation and insulin resistance, kaempferol treatment significantly reduced macrophage infiltration. This effect was mediated through the inhibition of LPS-induced inflammation via the STING/NLRP3/caspase-1 signaling pathway in RAW 264.7 macrophages, effectively alleviating inflammation and insulin resistance in the adipose tissue of db/db mice (57). Furthermore, in an LPS-induced sepsis model, kaempferol inhibited SphK1 expression in macrophages, thereby attenuating the inflammatory response and stabilizing the endothelial barrier through the SphK1/S1P signaling pathway (58). In the context of corneal transplantation, kaempferol suppressed NLRP3 inflammasome activation by inducing autophagy, which in turn inhibited macrophage polarization and ultimately alleviated corneal allograft rejection, improving transplantation success rates (52). In summary, macrophages serve as critical mediators of kaempferol’s actions, playing a central role in the regulation of immune responses. The ability of this compound to modulate macrophage polarization and function across diverse tissue and disease models underscores its potential in alleviating macrophage-associated systemic inflammation and brain inflammation, positioning it as a promising multi-target therapeutic agent for treating neuroinflammation and other immune-related disorders.

### 3.2 Microglia

Microglia, the tissue-resident macrophages of the central nervous system, serve as the principal immune guardians and are essential for sustaining cerebral homeostasis, modulating synaptic plasticity, and mediating responses to pathological insults (59, 60). Under physiological conditions, microglia contribute to neurodevelopmental processes, such as synaptic pruning and neuronal circuit refinement, through complement- and fractalkine-dependent mechanisms (61). In response to pathological stimuli, microglia undergo rapid activation, adopting either a pro-inflammatory (M1) or an anti-inflammatory (M2) phenotype. The balance between these phenotypes critically influences the course of neuroinflammation (62). While acute microglial activation promotes tissue repair and pathogen clearance, chronic activation drives sustained neuroinflammation through the excessive release of ROS, pro-inflammatory cytokines (e.g., TNF-α, IL-1β), and cytotoxic mediators, thereby exacerbating neurodegeneration in diseases such as AD and PD (63, 64). This chronically activated state establishes a self-sustaining cycle of inflammation and damage, making it a key therapeutic target in neurodegenerative diseases. In this context, immunomodulatory strategies targeting microglia show considerable promise. Existing research indicates that the natural flavonoid kaempferol exerts significant regulatory effects on microglia, the immune cells of the CNS. Studies have shown that kaempferol produces anti-inflammatory and neuroprotective effects by suppressing the overactivation of microglia, which is associated with neuroinflammation and neurodegenerative diseases like AD and PD (65). The underlying mechanisms of its action have been elucidated in detail across various disease models. In a model of spinal cord injury, pre-treatment with kaempferol alleviated microglia-mediated neuroinflammation following secondary injury by inhibiting the MAPKs-NF-κB signaling pathway and cellular pyroptosis (66). Regarding metabolic regulation, long-term administration of kaempferol reduced the density of activated microglia in the arcuate nucleus, leading to decreased body weight, reduced food intake, lower blood glucose levels, and improved insulin sensitivity, indicating its significant role in modulating systemic energy balance (67). These findings indicate that kaempferol modulates systemic metabolic homeostasis via central immune pathways. In the context of developmental neuropathology, administration of kaempferol in a neonatal rat model of cerebral palsy attenuated impairments in neuromotor development, hippocampal cell proliferation, and microglial activation. Additionally, it upregulated hippocampal gene expression of antioxidant enzymes, including superoxide dismutase (SOD) and catalase, underscoring its therapeutic potential to alleviate cerebral palsy-induced deficits in neuromotor behavioral development (68).

In the context of diabetic retinopathy (DR), kaempferol administration has been shown to markedly reduce inflammatory activity across *in vitro*, ex vivo, and *in vivo* models, primarily through modulation of immune cell responses. The compound suppresses pro-inflammatory signaling during DR progression by influencing microglia-mediated expression of arginase-1 and HO-1, thereby fostering an anti-inflammatory microenvironment (69). In the realm of neuropathic pain (NP), kaempferol was reported to attenuate the activation of the TLR4/NF-κB pathway in LPS-activated BV2 cells. This led to the inhibition of microglial activation and a shift from the M1 to the M2 phenotype, effectively alleviating pain behaviors (70). Similarly, in PD models, kaempferol exerted neuroprotective effects in 6-hydroxydopamine-induced PD rats and LPS-induced BV2 inflammatory cells by inhibiting the p38MAPK/NF-κB signaling pathway, thereby suppressing microglial pyroptosis and subsequent neuroinflammatory responses (71). In summary, microglia serve as key cellular mediators of kaempferol’s immunomodulatory actions, playing a central role in neuroimmune regulation. By modulating multiple critical signaling pathways, kaempferol balances microglial phenotypes and suppresses their overactivation, thereby disrupting the vicious cycle of neuroinflammation. These properties highlight its potential as a therapeutic agent for neuroinflammatory and neurodegenerative diseases, providing a theoretical basis for developing natural product-based neuroimmunomodulatory strategies.

### 3.3 T cells

**Figure 3:**
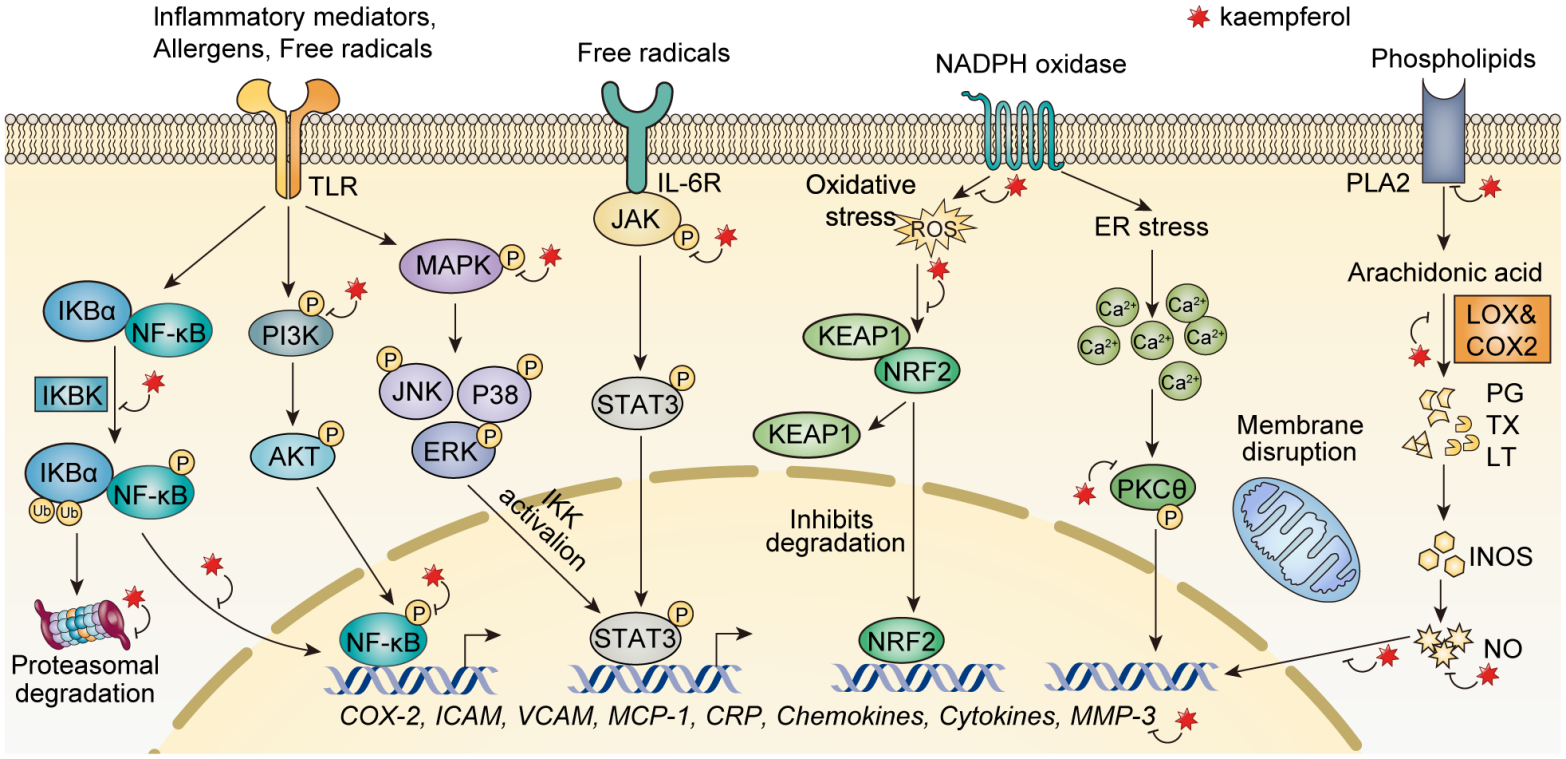
Mechanisms underlying the inhibition of neuroinflammation by kaempferol. Kaempferol attenuates neuroinflammation through coordinated regulation of key signaling pathways. It suppresses the activation of pro-inflammatory pathways, including NF-κB and JAK-STAT, thereby inhibiting the nuclear translocation of associated transcription factors and the subsequent expression of inflammatory mediators (e.g., TNF-α, IL-6). Concurrently, kaempferol activates the Nrf2 antioxidant pathway, promoting its nuclear translocation and the induction of cytoprotective enzymes (e.g., HO-1, NQO1). This dual regulatory action effectively reduces oxidative stress and dampens neuroinflammatory responses, highlighting kaempferol’s potential as a multi-target therapeutic agent for neurological disorders.

T cells play a critical role in the pathogenesis and progression of diverse neurological disorders, encompassing both autoimmune conditions and neurodegenerative diseases (72, 73). Within neurological diseases, autoreactive CD4+ T cells, notably Th1 and Th17 subsets, infiltrate the CNS via the BBB. Their subsequent release of pro-inflammatory mediators such as IFN-γ and IL-17 drives neuroinflammatory cascades, ultimately contributing to demyelination and axonal damage (74). Beyond autoimmunity, T cells also contribute to neurodegenerative diseases like AD and PD (75, 76). In AD, cytotoxic CD8+ T cells have been observed clustered around amyloid-β plaques, suggesting their direct involvement in neuroinflammation and neuronal loss through the release of cytotoxic mediators including perforin and granzymes (77). Similarly, in PD, α-synuclein-specific T cells may exacerbate dopaminergic neurodegeneration through sustained inflammatory responses, establishing an autoimmune neurodegenerative cycle (75). Notably, the role of T cells in the CNS demonstrates remarkable duality: in experimental stroke models, CD4+ T cells secreting anti-inflammatory cytokines (e.g., IL-4, IL-10) can mitigate neuroinflammation and promote tissue repair and functional recovery (78, 79). These dual effects underscore the complex interplay between T cell-mediated immunity and neural repair, offering potential therapeutic targets for precise modulation of neuroinflammation. Kaempferol, a natural compound with significant anti-inflammatory and immunomodulatory properties, plays an important role in treating inflammatory diseases. Research demonstrates that kaempferol exerts anti-inflammatory effects by inhibiting pyroptosis via regulation of the NLRP3/CASP1/GSDMD axis and suppressing hyperactive immune responses through modulation of T cell proliferation, thereby exhibiting therapeutic potential in rheumatoid arthritis (RA) (80). The mechanisms underlying these effects operate at multiple regulatory levels. Regarding T cell differentiation, kaempferol treatment in a murine model of rheumatoid arthritis suppressed the expression of RORγt and IL-17, while concurrently upregulating Foxp3, IL-10, and TGF-β. This modulation resulted in diminished production of pro-inflammatory cytokines and enhanced anti-inflammatory mediator release. Further mechanistic analysis revealed that kaempferol alleviates rheumatoid arthritis pathology in mice by modulating genes associated with the Treg/Th17 balance and attenuating cellular inflammation via the miR-34a/Foxp3 signaling axis (81). In an atopic dermatitis mouse model, kaempferol regulated T cell activation by inhibiting MRP-1 activity in activated T cells, demonstrating its protective role in T cell-mediated immune disorders (82). Regarding autoimmune regulation, kaempferol exhibits multifaceted therapeutic potential. In systemic vitiligo treatment, kaempferol-treated Tregs controlled the proliferation of CD8+ and CD4+ T cells and IFNγ production, consequently promoting melanocyte survival and proliferation (83). In transplantation tolerance, kaempferol promoted transplant tolerance in the presence of low-dose cyclosporine by enhancing Treg generation and reducing side effects, thereby blocking allograft rejection (84). During psoriasis treatment, kaempferol inhibited T cell proliferation and their mTOR signaling *in vitro*, reducing the percentage of IL-17A+ CD4+ T cells in the spleens and lymph nodes of psoriatic model mice, providing a theoretical basis for developing specific clinical treatments for psoriasis (85). In a rat arthritis model, kaempferol increased FOXP3 expression levels in Treg cells and prevented the progression of collagen-induced arthritis. Its unique anti-inflammatory mechanisms provide molecular insights into its anti-inflammatory effects in inflammatory diseases such as RA, systemic lupus erythematosus, and ankylosing spondylitis (86). In summary, these findings collectively demonstrate that T cells serve as crucial effector cells for kaempferol’s bioactivities. By modulating the differentiation, proliferation, and function of T cell subsets, kaempferol significantly promotes the restoration of immune homeostasis while exhibiting substantial therapeutic potential in alleviating both T cell-driven systemic inflammation and neuroinflammation. The precise regulatory effects of kaempferol on T cell-mediated immune responses position it as a promising natural compound for treating neuroinflammation-related diseases (**Figure 3**).

## 4 Kaempferol-mediated molecular mechanisms in neuroinflammation

### 4.1 Gut microbiota

A intricate bidirectional regulatory interaction, known as the “gut-brain axis,” operates between the gut microbiota and neuroinflammation. Evidence suggests that intestinal microbes modulate CNS inflammatory states through metabolites, immune modulation, and neuroendocrine signaling pathways (87). Specifically, gut microbiota-derived metabolites such as short-chain fatty acids, bile acids, and tryptophan metabolites not only contribute to maintaining intestinal barrier integrity but can also reach the CNS via the circulatory system, directly or indirectly regulating microglial activation and function, thereby playing a key role in the initiation and progression of neuroinflammation.

In a murine model of ulcerative colitis, kaempferol treatment effectively improved gut microbial composition, increased the Firmicutes/Bacteroidetes ratio, and reshaped the gut microbiome structure. It significantly elevated the linear discriminant analysis scores of beneficial bacteria (e.g., Prevotellaceae and Ruminococcaceae) and reduced the relative abundance of the conditional pathogen Proteobacteria. This optimization of the microbial community structure was accompanied by decreased activity of TLR4-related signaling pathways, thereby modulating the gut microbiota and TLR4 signaling to alleviate the inflammatory response and exert a protective effect against ulcerative colitis (34). In obesity-related disorders, exogenous kaempferol supplementation modulated cecal microbial ecology, restored gut microbiota diversity, and reduced the activation of the TLR4/NF-κB pathway, thereby improving intestinal barrier integrity, suppressing intestinal inflammation, and conferring anti-obesity effects (56). This mechanism involves kaempferol’s promotion of intestinal tight junction protein expression, enhancing barrier function and preventing inflammatory factors like bacterial LPS from entering the circulation. It has been reported that kaempferol treatment exerts anti-arthritic effects by reversing perturbations in metabolites involved in energy production and the metabolism of tryptophan, fatty acids, and secondary bile acids in the gut contents of an arthritis mouse model, restoring the normal metabolic profile (69). This suggests kaempferol not only regulates microbial composition but also influences the metabolic functions of the microbiota to exert its therapeutic effects. Recent research further expands our understanding of the kaempferol-microbiota axis. Kaempferol treatment was reported to increase the abundance of microbial species with anti-cancer properties while reducing species associated with inflammation, obesity, and metabolic disorders. This subsequently inhibited bile acid synthesis, increased G protein-coupled receptor activity, and decreased NOD-like receptor activity. This multi-level regulation ultimately suppressed tumor progression (33). Notably, these microbial changes were closely linked to the modulation of inflammatory signaling pathways, suggesting kaempferol may regulate systemic inflammatory status by remodeling the gut microecology. 4-Hydroxyphenylacetic acid (4-HPCA), a major intestinal metabolite of kaempferol, exerts potent antibacterial activity against Listeria monocytogenes in a dose-dependent manner by compromising cell membrane integrity and altering membrane potential. Additionally, 4-HPCA significantly downregulates the expression of key virulence genes (hlyA, prfA, and inlA), collectively leading to bacterial cell death (88). Kaempferol, as one of four flavonoid inhibitors, significantly enhances the bidirectional uptake of dihydroquercetin (DHQ) and improves its absorption by downregulating P-glycoprotein (P-gp) mRNA and protein expression in KB/MDR1 and Caco-2 cell models (89). In summary, kaempferol modulates the composition and function of the gut microbiota, alters the profile of microbial metabolites, and thereby exerts therapeutic effects in neuroinflammation-related diseases through multiple communication pathways of the gut-brain axis. This mechanism of indirectly influencing neuroinflammation by regulating the gut microbiota provides a new perspective for understanding kaempferol’s multi-target actions and offers a theoretical basis for its clinical application in neurodegenerative and neuroinflammation-related diseases.

### 4.2 Kaempferol as an inflammatory factor modulator

#### 4.2.1 Inhibition of the MAPK signaling pathway

The MAPK signaling cascade serves a pivotal function in the initiation, perpetuation, and termination of inflammatory responses through its precise control over the transcription and expression of pro-inflammatory cytokines, chemokines, and associated inflammatory mediators (90, 91). The MAPK signaling network comprises three main subfamilies: Extracellular Signal-Regulated Kinase 1/2, c-Jun N-terminal Kinase (JNK), and p38 MAPK. These kinases can be activated by various endogenous and exogenous stimuli, including danger signals such as Pathogen-Associated Molecular Patterns (PAMPs) and DAMPs (92, 93). Upon phosphorylation and activation, these kinases regulate downstream key transcription factors like NF-κB and AP-1 via cascade reactions, significantly enhancing the expression of numerous inflammation-related genes (94, 95). Notably, p38 MAPK holds a unique position in the biosynthesis of core pro-inflammatory cytokines such as TNF-α, IL-1β, and IL-6, which play decisive roles in the pathogenesis of neuroinflammation and related neurological disorders (96). Concurrently, the JNK and Extracellular Signal-Regulated Kinase (ERK) pathways further amplify and sustain the inflammatory response by regulating processes like cell proliferation, apoptotic balance, and immune cell differentiation (97, 98). Given the pivotal role of the MAPK pathway in inflammatory signal transduction and its dysregulation in various pathological states, it represents an important target for anti-inflammatory drug development. Recent studies have revealed that the natural flavonoid kaempferol exerts multi-faceted regulatory effects on the MAPK signaling pathway. In the cardiovascular field, kaempferol significantly suppressed the activation of the MAPK/NF-κB signaling axis and upregulated the Nrf2/HO-1 pathway in an atherosclerosis model, effectively inhibiting inflammation and delaying the formation and progression of atherosclerotic plaques (11). Notably, kaempferol’s regulation of different MAPK isoforms is tissue-specific. In an oral cancer model, kaempferol selectively downregulated phosphorylated ERK levels while enhancing the activity of phosphorylated JNK (p-JNK) and phosphorylated p38 kinase, promoting tumor cell apoptosis and autophagy through this differential regulation and inhibiting tumor progression (99). In skeletal system diseases, kaempferol demonstrates bidirectional regulatory capabilities. In an osteoporosis model, it promoted JNK and p38-MAPK expression, counteracting dexamethasone’s inhibition of osteogenic function (100); whereas in an intervertebral disc degeneration model, it inhibited p38 MAPK pathway phosphorylation, reducing local inflammation and delaying disc degeneration (101). Similarly, in a periprosthetic osteolysis model, kaempferol effectively suppressed osteoclast differentiation and function by downregulating JNK and p38-MAPK expression (102). Regarding inflammatory diseases, kaempferol exhibits significant anti-inflammatory properties. It was shown to reduce the production of inflammatory mediators like IL-1β, TNF-α, and MIP-2 by decreasing phosphorylated p38 MAPK expression, thereby reducing macrophage and neutrophil recruitment and promoting the healing of fungal keratitis (103). In a drug-induced renal injury model, kaempferol inhibited MAPK pathway activation, increased renal glutathione (GSH) levels and SOD activity, lowered malondialdehyde (MDA) content, and ameliorated doxorubicin-induced kidney injury (104). In an LPS-induced inflammatory model in gallbladder epithelial cells, kaempferol effectively mitigated the inflammatory response by inhibiting MAPK/NF-κB signaling (105). Of particular interest is kaempferol’s protective role in neurological diseases. In AD research, kaempferol enhanced neuronal resistance to Aβ toxicity by modulating the ER/ERK/MAPK signaling pathway (106). A recent study also found that kaempferol synergistically activated both the Phosphatidylinositol 3-kinase(PI3K)/Protein Kinase B(AKT) and MAPK signaling pathways in C2C12 cells, promoting glucose uptake, mitochondrial biogenesis, and protein synthesis, thereby improving exercise performance and alleviating physical fatigue (107). These findings indicate that kaempferol exerts therapeutic effects in various disease models through the specific regulation of different components of the MAPK signaling pathway. Its precise regulatory capacity over the MAPK network, particularly its key role in neuroinflammation, provides a new theoretical basis and research direction for developing natural product-based strategies for treating neuroinflammation-related diseases. The unique value of kaempferol as a multi-target natural compound in regulating the MAPK pathway warrants further in-depth investigation.

#### 4.2.2 Inhibition of the PI3K/AKT signaling pathway

As a critical intracellular signaling hub, the PI3K/AKT pathway exerts precise regulatory control over neuroinflammatory processes by modulating glial cell activation states, balancing cytokine production, and determining neuronal survival outcomes (2, 108, 109). Upon activation by extracellular stimuli such as pro-inflammatory cytokines (e.g., TNF-α, IL-1β) or growth factors, PI3K is recruited to the plasma membrane where it catalyzes the phosphorylation of phosphatidylinositol bisphosphate (PIP2) to generate phosphatidylinositol trisphosphate (PIP3). This promotes AKT recruitment to the membrane and its full activation via PDK1-mediated phosphorylation at Thr308 (110, 111). In microglia and astrocytes, activated AKT can effectively suppress NF-κB-driven neuroinflammatory responses either by directly phosphorylating the IKK complex or by enhancing the expression of anti-inflammatory mediators like IL-10 (112, 113). Furthermore, AKT regulates neuronal survival signaling networks by phosphorylating downstream key targets such as Glycogen synthase kinase-3 beta, thereby significantly mitigating neuroinflammation-associated apoptosis (114–116). Dysregulation of this pathway is closely linked to various neurological diseases, including AD, PD, and MS, highlighting its considerable potential as a therapeutic target for neuroinflammatory diseases (117). Recent research has found that the natural flavonoid kaempferol exerts multi-level regulatory effects on the PI3K/AKT pathway. In cardiovascular disease, kaempferol can activate the PI3K/AKT/Nrf2 signaling pathway by upregulating the G protein-coupled estrogen receptor, significantly alleviating atherosclerotic lesions (118). Similarly, in myocardial ischemia-reperfusion (I/R) injury, kaempferol exerted notable protective effects against hypoxia/reoxygenation-induced cardiomyocyte damage by promoting the expression of the Notch/PTEN/Akt signaling pathway (119). In metabolic diseases, kaempferol effectively stimulated AKT signaling upregulation in a type 2 diabetes model, demonstrating therapeutic potential by modulating insulin signaling and improving glucose metabolic homeostasis (120). In pulmonary arterial hypertension pathogenesis, kaempferol treatment reduced the phosphorylation levels of AKT and GSK3β while inhibiting the expression of CDK2, CDK4, and BCL-2, effectively suppressing abnormal proliferation and promoting apoptosis of pulmonary arterial smooth muscle cells, thereby ameliorating the pathological progression in rats (36940862). In exercise physiology, kaempferol concurrently activated both PI3K/AKT and MAPK signaling pathways, promoting protein synthesis and glucose uptake while reducing ROS levels, playing an important role in improving exercise performance and alleviating physical fatigue (107). Further research confirmed that kaempferol enhanced the migration and differentiation capabilities of C2C12 myoblasts by regulating the ITG1B/FAK/Paxillin and IGF1R/AKT/mTOR pathways, providing molecular-level evidence for maintaining muscle health (121). In cancer research, kaempferol demonstrated significant anti-cancer activity in various models including gastric, cervical, pancreatic, and hepatocellular cancers. The mechanisms involve inhibiting the PI3K/AKT/mTOR or AKT/GSK3β signaling pathways, thereby inducing tumor cell apoptosis and autophagy (122–125). In inflammatory diseases, kaempferol exhibited marked anti-inflammatory effects in LPS- and TNF-α-induced inflammatory environments. It effectively inhibited abnormal angiogenesis in intestinal microvascular endothelial cells by suppressing the VEGF/Akt/p38 signaling pathway and maintaining intestinal vascular barrier integrity (126). A recent study showed that kaempferol could rescue the osteogenic differentiation capacity of periodontal ligament stem cells (PDLSCs) and alleviate bone loss in a periodontitis microenvironment by targeting EphrinB2 and simultaneously activating both the PI3K/Akt and P38 signaling pathways (127). Particularly noteworthy is that in neurological disease research, kaempferol has been proven to effectively inhibit neurodegeneration and neuroinflammatory responses while promoting neurogenesis and improving cognitive function by regulating the PI3K/Akt pathway (128). Based on this evidence, it can be inferred that the PI3K/AKT signaling pathway is a key molecular target mediating kaempferol’s anti-inflammatory effects, playing an important role in the treatment of neuroinflammation-related diseases. Kaempferol’s multi-target, multi-level regulation of the PI3K/AKT signaling network demonstrates its great potential for development as a therapeutic agent for neuroinflammation.

#### 4.2.3 Inhibition of the JAK/STAT signaling pathway

The Janus kinase (JAK)/Signal Transducer and Activator of Transcription (STAT) signaling pathway serves as a core mediator of cytokine signal transduction, playing a critical role in mediating neuroinflammatory responses and widely participating in the pathogenesis of various neurological disorders, including AD, PD, MS, and ischemic stroke (129, 130). The activation of this pathway is initiated upon cytokine engagement with their cognate receptors. Specifically, when stimulated by pro-inflammatory mediators such as IL-6, IFN-γ, and TNF-α, receptor-associated JAKs undergo autophosphorylation and subsequently catalyze the phosphorylation of specific tyrosine residues on STAT proteins. This post-translational modification promotes STAT dimerization and translocation to the nucleus, where the dimers bind to defined DNA sequences within the promoter regions of target genes. Through this mechanism, the pathway exerts precise transcriptional control over genes involved in inflammation, glial activation, and neuronal survival (131, 132). Notably, persistent dysregulation of the JAK/STAT pathway forms a positive feedback loop with excessive microglial and astrocytic activation, leading to a sustained neuroinflammatory microenvironment and progressive neuronal damage (133–135). Consequently, pharmacological inhibition of JAK/STAT signaling has shown significant neuroprotective effects in preclinical models of various neurological diseases, effectively reducing neuroinflammation and improving cognitive and motor behavioral performance (136). Recent studies indicate that the natural flavonoid kaempferol has a significant inhibitory effect on the JAK/STAT signaling pathway. In skin immunology research, kaempferol treatment effectively suppressed the abnormal activation of the pro-inflammatory JAK/STAT pathway, reduced the expression levels of key inflammatory cytokines such as IL-23, IL-17A, TNF-α, IL-6, and IL-1β, and consequently markedly improved imiquimod-induced psoriasis-like skin inflammation (137). In the regulation of the tumor immune microenvironment, kaempferol precisely modulated JAK-STAT3 pathway activity, effectively inhibiting tumor immune evasion mechanisms, while also significantly suppressing tumor angiogenesis and metastatic potential by downregulating the expression of matrix metalloproteinases like MMP-3 and MMP-9 (138). In liver disease research, kaempferol has been identified as a promising therapeutic agent. It exerts multi-target inhibition of the JAK-STAT pathway, concurrently providing anti-inflammatory, anti-fibrotic, and anti-pro-inflammatory gene expression effects, effectively improving immune-inflammatory responses in the liver microenvironment, reducing hepatocyte apoptosis, and offering comprehensive hepatoprotection (139). In the treatment of digestive system diseases, kaempferol significantly reduced the expression of pro-inflammatory factors like IL-6 and IL-1β by modulating JAK/STAT pathway activity, thereby effectively treating chronic atrophic gastritis and promoting gastric mucosal repair (140). Based on this evidence, it can be concluded that kaempferol plays a key role in regulating neuroinflammatory processes by specifically inhibiting the abnormal activation of the JAK/STAT signaling pathway. The elucidation of this molecular mechanism not only deepens our understanding of kaempferol’s pharmacological actions but also provides a new molecular target and a solid theoretical basis for developing treatment strategies for neuroinflammation-related diseases. As a naturally derived JAK/STAT pathway modulator, kaempferol shows broad application prospects in the prevention and treatment of neurological diseases.

#### 4.2.4 Inhibition of PKC activity

Protein Kinase C (PKC) is a family of widely expressed serine/threonine kinases that play a central role in cellular signaling networks, precisely regulating various physiological processes including cell proliferation, differentiation, migration, and apoptosis (141, 142). Family members are classified into three major subclasses based on structure and activation mechanisms: classical (cPKC), novel (nPKC), and atypical (aPKC), each with specific tissue distribution and functions. Accumulating evidence indicates that specific PKC isoforms play crucial roles in regulating neuroinflammatory responses (143). The pathological features of neuroinflammation primarily include abnormal activation of microglia and astrocytes, and excessive release of pro-inflammatory cytokines (e.g., TNF-α, IL-1β, IL-6), which collectively lead to neuronal damage and synaptic dysfunction (144). Recent studies suggest PKC activation can have opposing effects depending on context: it may exacerbate neuroinflammation by promoting inflammatory mediator production (143, 145), or exert protective effects under specific conditions. In an experimental MS model, TPPB alleviated neuroinflammation by modulating PKC activity, promoting CNS regeneration and repair, and ultimately improving neurodegenerative progression (146). Research shows kaempferol has significant regulatory effects on the PKC signaling pathway. In vascular function regulation, kaempferol inhibited PKC activity by suppressing PKC-potentiated protein phosphatase inhibitor-17, thereby improving vascular contractility (147). In immunomodulation, kaempferol showed significant activity in IgE-mediated allergic reactions. It binds to the antioxidant protein DJ-1, inhibits its translocation process, and consequently inhibits the activation of the PKC signaling pathway, thus preventing mast cell-mediated allergic diseases (148). Recent research further reveals kaempferol’s important role in neurological diseases. In a neuroinflammatory disease model, *Tetrastigma hemsleyanum* Diels et Gilg, with kaempferol as a main active component, significantly reduced levels of pro-inflammatory cytokines like IL-1β, IL-6, and TNF-α by inhibiting the PKC-δ/caspase-1 signaling pathway, thereby suppressing neuroinflammation and exerting anticonvulsant effects (149). Based on this evidence, it is plausible to infer that PKC may serve as a key signaling molecule mediating kaempferol’s anti-inflammatory effects, a mechanism with significant therapeutic potential in the pathophysiology of neuroinflammation-related diseases. Concurrently, Nrf2 also plays a central regulatory role at the molecular level. By binding to the Antioxidant Response Element (ARE), it activates the expression of a suite of cytoprotective genes. Kaempferol regulates Nrf2 activation through specific signal transduction pathways, thereby mediating its anti-inflammatory and antioxidant biological effects. This intricate molecular regulatory mechanism demonstrates significant intervention potential and therapeutic promise, particularly in the synergistic control of oxidative stress and inflammatory responses within the pathology of neuroinflammation-related diseases, providing a new theoretical basis for developing natural product-based neuroprotective strategies.

#### 4.2.5 Activation of the Nrf2 signaling pathway

The transcription factor Nrf2 is a master regulator of cellular defense against oxidative stress, exerting its cytoprotective functions by coordinately regulating the expression of a battery of genes driven by the ARE. These key genes include HO-1, NADPH quinone dehydrogenase 1, and GSH S-transferases (150). Recent research indicates that Nrf2 activation acts not only through classic antioxidant pathways but also exerts powerful anti-neuroinflammatory effects by inhibiting excessive microglial activation and interfering with NF-κB-mediated inflammatory signaling (151, 152). Preclinical studies further confirm that Nrf2 gene deletion significantly exacerbates neuroinflammation, whereas pharmacological activation of the Nrf2 pathway effectively reduces neuroinflammation and enhances neuronal survival (153, 154). Furthermore, the complex interplay between Nrf2 and other neuroprotective pathways, such as the autophagy-lysosome system, suggests its regulatory role in neuroinflammatory diseases is broader and more intricate than previously recognized (155, 156). Given its central position in coordinately mitigating oxidative stress and inflammation, therapeutic strategies targeting the Nrf2 pathway hold significant promise for neuroinflammation and neurodegenerative diseases (157, 158). Studies demonstrate that the natural flavonoid kaempferol has multifaceted activating effects on the Nrf2 signaling pathway. In a stroke model, kaempferol activated the Nrf2/SLC7A11/GPX4 pathway, inhibited apoptosis, thereby alleviating I/R injury and providing effective neuroprotection post-stroke (159). In cardiovascular disease, kaempferol reduced myocardial injury and improved cardiac function in a myocardial I/R model by increasing Nrf2 acetylation (160); in atherosclerosis, kaempferol inhibited foam cell formation and delayed disease progression by coordinately regulating the NF-κB/MAPK and HO-1/Nrf2 pathways (11); in doxorubicin-induced myocardial injury, kaempferol promoted Nrf2 protein accumulation and nuclear translocation, inhibiting mitochondrial ROS-dependent ferroptosis and providing cardioprotection (161). Additionally, kaempferol exerted anti-atherosclerotic effects by upregulating the PI3K/AKT/Nrf2 pathway (118). A recent study also found kaempferol alleviated LPS-induced decreases in cell viability and LDH release, inhibited excessive ROS generation, thereby reducing oxidative stress and macrophage pyroptosis, and suppressing atherosclerosis progression via NRF2 pathway activation (162). In vascular biology, kaempferol promoted Nrf2/HO-1 protein expression levels and suppressed TNF-α, IL-6 levels, and NF-κB activation in aortic tissue and HUVECs, protecting vessels from oxidative stress and inflammation-induced damage (163). In liver disease, kaempferol acts through multiple pathways: it activated the Nrf2 pathway and upregulated Gpx4 in mouse liver and L02 cells to inhibit acetaminophen-induced ferroptosis (164); it exerted anti-inflammatory and antioxidant effects by upregulating the Nrf2/HO-1 axis and inhibiting NF-κB p65 interaction with Keap1 (165); it increased Nrf2 transactivation in hepatocytes, lowered ROS and MDA levels, promoted the activation of β-oxidation related genes (CPT-1, PPAR-α), and reduced cellular lipid accumulation (166). Recent studies also show kaempferol exerted antioxidant, anti-inflammatory, and hepatoprotective effects by inhibiting the MAPK/NF-κB pathway and activating the AMPK/Nrf2 pathway (167). In a hepatic I/R injury model, kaempferol activated the Nrf2/HO-1 pathway, inhibiting oxidative stress and inflammation and alleviating liver damage (168). In respiratory disease, kaempferol-mediated activation of the NOX/ROS/c-Src/Pyk2/PKCα/p38α MAPK and JNK1/2 signaling cascades led to Nrf2 activation, promoting its binding to the ARE on the HO-1 promoter and inducing HO-1 expression, thereby inhibiting LPS-mediated inflammation in human alveolar epithelial cells and preventing lung inflammation (169). In reproductive system research, kaempferol alleviated age-related ovarian reserve reduction by inhibiting HSP90 expression and upregulating NRf2, exerting antioxidant effects (170). In metabolic disease, kaempferol upregulated Nrf-2/HO-1 in a diabetic nephropathy model, exerting antioxidant effects while also reducing fasting blood glucose, increasing fasting insulin levels, and preventing diabetic kidney disease progression (171). In ophthalmology, kaempferol treatment promoted SIRT1 nuclear accumulation and activated nuclear Nrf2 levels, exerting antioxidant and anti-inflammatory effects and preventing H_2_O_2_-induced inflammation and apoptosis in retinal pigment epithelial cells (172). These collective results demonstrate that kaempferol exerts broad antioxidant and anti-inflammatory effects across different organ systems through multi-target, multi-level activation of the Nrf2 signaling pathway. Particularly in neuroinflammation-related diseases, kaempferol’s specific ability to activate the Nrf2 pathway provides a solid molecular foundation for its potential in treating neurodegenerative diseases, showcasing significant clinical application prospects.

#### 4.2.6 Inhibition of the NF-κB signaling pathway

The NF-κB signaling pathway is a core regulatory hub for inflammatory responses and plays a widespread and critical role in the pathogenesis of neuroinflammatory diseases (173, 174). NF-κB is a highly conserved family of transcription factors, including subunits like RelA (p65), RelB, c-Rel, p50, and p52, which precisely coordinate the expression of numerous inflammation-related genes, including pro-inflammatory cytokines, chemokines, and adhesion molecules, by forming homo- or heterodimers (175, 176). In the CNS, aberrant NF-κB activation in microglia, astrocytes, and neurons creates a positive feedback loop that persistently exacerbates neuroinflammation, thereby significantly promoting the pathology of neurodegenerative diseases such as AD, PD, and MS (136, 151). Specifically, sustained NF-κB activation promotes the excessive release of inflammatory mediators like TNF-α, IL-6, and inducible nitric oxide synthase (iNOS). These mediators ultimately lead to neuronal damage and exacerbated neurotoxicity through various mechanisms, including disrupting neuronal calcium homeostasis, impairing mitochondrial function, and causing synaptic dysfunction (151, 177). Emerging evidence suggests that precise intervention targeting the NF-κB pathway holds significant therapeutic potential for mitigating neuroinflammatory damage. For instance, specific knockout of key NF-κB subunits in microglia significantly reduced neuroinflammation and improved neurological outcomes in experimental neurodegenerative models (178, 179). Research demonstrates that the natural flavonoid kaempferol exerts multi-level inhibitory effects on the NF-κB signaling pathway. In an intestinal inflammation model, kaempferol inhibited key nodes of the TLR4-NF-κB pathway, significantly increased the expression of tight junction proteins ZO-1, occludin, and claudin-1, while reducing the release of pro-inflammatory factors like IL-1β, IL-6, and TNF-α. This effectively prevented dextran sulfate sodium-induced intestinal barrier disruption and exerted a protective effect in colitis mice by modulating gut microbiota balance (34). In diabetes-associated intestinal vascular pathology, kaempferol prevented hyperglycemia-induced overproduction of ICAM-1 and VCAM-1 by inhibiting NF-κB p65 nuclear translocation, thereby suppressing intestinal inflammation and protecting intestinal vascular barrier function (180). In cardiovascular disease, kaempferol exhibits multi-target protective effects. It regulated CD36-mediated mitochondrial ROS production by inhibiting the Piezo1 channel and calcium influx, subsequently modulating downstream pathways including NF-κB/MAPK and HO-1/Nrf2, inhibiting foam cell formation, and delaying atherosclerosis progression (11). Concurrently, kaempferol significantly reduced cardiomyocyte apoptosis and inflammation, mitigating myocardial injury by modulating the STING/NF-κB pathway (181). In neurological diseases, kaempferol inhibited the activation of the TLR4/NF-κB pathway in microglia, promoting a shift from the pro-inflammatory M1 phenotype to the anti-inflammatory M2 phenotype, effectively alleviating NP (70). In osteoporosis prevention, kaempferol alleviated oxidative stress via the SRC/NF-κB-AKT/NOS3 axis, preventing osteoclast formation and bone resorptive activity (182). In hepatoprotection, kaempferol exerted synergistic antioxidant and anti-inflammatory effects by upregulating the Nrf2/HO-1 axis and inhibiting the interaction between NF-κB p65 and Keap1, effectively protecting the liver from injury (165, 167). Similarly, in a renal disease model, kaempferol exerted anti-inflammatory and antioxidant effects by inhibiting NF-κB p65 activity and promoting Nrf2 nuclear translocation, improving pathological changes in rat nephropathy (165). In the peripheral nervous system, kaempferol significantly attenuated bupivacaine-induced neurotoxicity in dorsal root ganglion neurons by mitigating bupivacaine-induced upregulation of TNF receptor-associated factor 6 and subsequent NF-κB activation (183). In metabolic disease research, kaempferol supplementation improved intestinal barrier integrity and suppressed intestinal inflammation by reducing TLR4/NF-κB pathway activation, thereby exerting anti-obesity effects (56). A recent study also found that kaempferol significantly alleviated pathological changes in hyperuricemia combined with gouty arthritis by regulating urate transporters and the NLRP3/NF-κB signaling pathway (184). In summary, kaempferol plays a key regulatory role in the neuroinflammatory process by inhibiting NF-κB pathway activation through multiple routes. These findings not only deepen our understanding of kaempferol’s pharmacological mechanisms but also provide an important theoretical basis and new research directions for developing natural product-based strategies for treating neuroinflammation. As a multi-target NF-κB pathway modulator, kaempferol shows broad application prospects in the prevention and treatment of neuroinflammation-related diseases.

### 4.3 Kaempferol as an anti-oxidant

Oxidative stress is a pathophysiological state resulting from an imbalance between the generation of ROS and the cellular antioxidant defense mechanisms. It plays a pivotal role in the initiation and progression of neuroinflammation and neurodegenerative diseases (185). Under normal physiological conditions, ROS serve as crucial second messengers involved in regulating vital processes such as cell proliferation, differentiation, and apoptosis. However, under various pathological stimuli, excessive ROS accumulation can overwhelm the cellular clearance capacity, leading to irreversible damage including lipid peroxidation, protein denaturation/inactivation, and DNA oxidative damage, thereby further exacerbating neuroinflammatory responses (186, 187). Neuroinflammation, driven by aberrant activation of microglia and astrocytes and the excessive release of pro-inflammatory cytokines such as IL-6 and TNF-α, forms a tightly coupled positive feedback loop with oxidative stress. This self-perpetuating vicious cycle significantly aggravates neuronal damage and synaptic dysfunction (174, 188, 189). Recent evidence indicates that oxidative stress activates several key inflammatory signaling pathways, including NF-κB and the NLRP3 inflammasome, thereby exacerbating the neuroinflammatory cascade in neurodegenerative conditions like AD and PD by promoting the transcription and release of inflammatory mediators (190). Research demonstrates that kaempferol, as a natural antioxidant, exhibits significant regulatory effects on oxidative stress in various disease models. In the cardiovascular field, kaempferol plays an important protective role in the treatment of myocardial injury. It activates the NRF2/SLC7A11/GPX4 signaling axis, inhibits mitochondrial ROS-dependent ferroptosis, and effectively prevents doxorubicin-induced myocardial damage (161). Concurrently, kaempferol inhibits inflammatory responses through multi-target actions, including downregulating CD36 expression, stabilizing mitochondrial membrane potential, reducing ROS production, inhibiting MAPK/NF-κB pathway activation, blocking Ca²^+^ influx, and significantly elevating Nrf2/HO-1 levels in macrophages, thereby suppressing the formation and progression of atherosclerotic plaques (11). In a rat myocardial ischemia model, kaempferol pretreatment effectively reduced MDA concentration and enhanced SOD activity. By promoting Nrf2 acetylation, it protected myocardial mitochondrial structural integrity and significantly alleviated oxidative stress injury induced by myocardial ischemia (189). In liver disease research, kaempferol’s regulatory effect on ferroptosis is particularly prominent. Ferroptosis, an iron-dependent form of cell death associated with lipid peroxide accumulation, is closely linked to various liver injuries. Kaempferol activates the Nrf2 signaling pathway in mouse hepatocytes, upregulates Gpx4 expression, effectively inhibits acetaminophen-induced ferroptosis, and alleviates chemical liver injury (164). In renal protection, kaempferol significantly ameliorates doxorubicin-induced kidney injury, manifested by reduced proteinuria and improved renal function. The mechanism involves restoring renal GSH content, increasing SOD and catalase activities, inhibiting excessive MDA production, and reducing ROS generation by suppressing MAPK pathway activation (104). In respiratory diseases, kaempferol exerts anti-inflammatory effects by inducing the expression of HO-1. The upregulation of this key antioxidant enzyme is closely associated with the inhibition of ROS production, providing a new therapeutic avenue for acute lung injury (169). In oncology research, kaempferol displays dual regulatory characteristics: in pancreatic cancer, it increases ROS levels via the Akt/mTOR pathway to promote tumor cell apoptosis (125); whereas in models of hepatocellular carcinoma, prostate cancer, and breast cancer, kaempferol primarily exerts anti-cancer effects through ROS inhibition mechanisms (191, 192). Recent studies have also developed mitochondria-targeted kaempferol nanoparticles. This innovative delivery system synergistically improves mitochondrial homeostasis, redox balance, energy metabolism, and inflammatory responses by promoting mitophagy in severe acute pancreatitis, demonstrating promising therapeutic potential (193, 194). In summary, kaempferol effectively disrupts the vicious cycle of neuroinflammation by multi-faceted modulation of the cross-talk between oxidative stress and inflammatory responses. These findings suggest that kaempferol treatment holds promise for reducing the severity of neuroinflammation by inhibiting oxidative stress, thereby providing effective protection for the nervous system and offering new strategies and a theoretical basis for the prevention and treatment of neurodegenerative diseases.

### 4.4 Kaempferol as a modulator of pro-inflammatory enzyme activity

Neuroinflammation is a complex pathological response involving multiple cellular and molecular events, characterized primarily by aberrant activation of glial cells (including microglia and astrocytes) and the release of a series of pro-inflammatory mediators (144). The core molecular mechanisms of this process involve the upregulated expression of various inflammation-associated enzymes, among which cyclooxygenase (COX) and iNOS are of particular interest due to their key roles in initiating and amplifying the neuroinflammatory cascade (195). COX, particularly its inducible COX-2 isoform, catalyzes the conversion of arachidonic acid to prostaglandins (e.g., PGE2), lipid mediators that contribute to altered BBB permeability, neuronal damage, and synaptic dysfunction (196). Simultaneously, iNOS overexpression leads to excessive NO production. NO reacts with superoxide anion to form peroxynitrite, causing protein nitration, lipid peroxidation, and DNA damage, thereby exacerbating oxidative stress and neurotoxicity (197). Emerging evidence indicates a synergistic interaction between COX and iNOS, which significantly aggravates the neuroinflammatory process as they collectively promote ROS generation and pro-inflammatory cytokine release (198, 199). In activated microglia, COX-2 and iNOS are often co-induced by pro-inflammatory stimuli such as LPS and Aβ, establishing a self-sustaining deleterious feedback loop that perpetuates chronic neuroinflammation and promotes neurodegeneration (200, 201). Research demonstrates that the natural flavonoid kaempferol exerts significant regulatory effects on COX- and iNOS-mediated inflammatory pathways. In neurological disease-related research, kaempferol was reported to reverse increased IL-1β and IL-6 expression and promote TGF-β1 and IL-10 expression by inhibiting the COX-2/PGE2 signaling pathway—which regulates neuroinflammation, neurotransmitter imbalance, and neurogenesis deficits—thereby ameliorating the pathological process of breast cancer-related depression and reducing its recurrence rate and mortality (9). In a prostate disease model, kaempferol treatment effectively reversed LPS-induced increases in COX-2 and iNOS levels, reduced mitochondrial ROS production, alleviated LPS-induced mitochondrial ultrastructural damage in prostate tissue, and inhibited the progression of prostate hypertrophy and cancer (191). In the field of metabolic diseases, kaempferol exhibits specific inhibitory effects on the iNOS pathway. Studies indicate that pro-inflammatory cytokines and NO play crucial roles in pancreatic β-cell dysfunction. Kaempferol treatment significantly inhibited NO generation, iNOS protein and mRNA levels, decreased iNOS protein stability, and directly inhibited NOS enzyme activity, all contributing to its beneficial effects in diabetes treatment (202). In liver disease research, where the interaction between oxidative stress and inflammation is critical, kaempferol treatment exerted synergistic antioxidant and anti-inflammatory effects by reducing pro-inflammatory mediators TNF-α and IL-1β, as well as COX-2 and iNOS expression levels, effectively protecting hepatocytes from injury (167). In summary, kaempferol acts as a multi-target modulator of pro-inflammatory enzyme activity, demonstrating significant antioxidant and anti-inflammatory effects at the molecular level by specifically regulating key pro-inflammatory enzymes such as COX-2 and iNOS. This dual inhibitory capacity enables it to effectively disrupt the self-sustaining cycle of neuroinflammation, showcasing broad interventional value and therapeutic application prospects under various pathological conditions. The precise regulatory properties of kaempferol on the inflammatory enzyme network makes it a promising candidate molecule for research into the prevention and treatment of inflammation-related diseases, holding particular value in developing therapeutic strategies for neuroinflammatory disorders.

### 4.5 Enhancement of autophagy

Autophagy is a highly conserved lysosomal degradation pathway crucial for clearing damaged organelles, misfolded protein aggregates, and intracellular pathogens, thereby maintaining intracellular homeostasis and ensuring cell survival under metabolic stress and oxidative conditions (203). New evidence reveals a complex and finely tuned bidirectional regulatory relationship between neuroinflammation and autophagy. Dysregulated autophagy is believed to significantly exacerbate neuroinflammatory responses, while persistent excessive inflammation can further impair autophagic processes, forming a vicious cycle that promotes neurodegeneration (204–206). For instance, impaired autophagy leads to abnormal intracellular accumulation of misfolded proteins (e.g., Aβ, α-synuclein). These aggregates trigger downstream inflammatory cascades by activating the NLRP3 inflammasome, amplifying neuroinflammatory signals (205, 207). Conversely, pro-inflammatory cytokines like TNF-α have been shown to dynamically regulate autophagy, potentially inducing it as a protective mechanism in acute phases while suppressing it under chronic conditions (2). Recent studies highlight the significant therapeutic potential of pharmacologically targeting autophagy to alleviate neuroinflammation. Pharmacological interventions inducing autophagy via mTOR inhibition or AMPK activation have shown notable neuroprotective effects in various neuroinflammatory disease models (208), underscoring the therapeutic value of modulating autophagy to break the harmful feedback loop between autophagic dysfunction and neuroinflammation.

**Figure 4:**
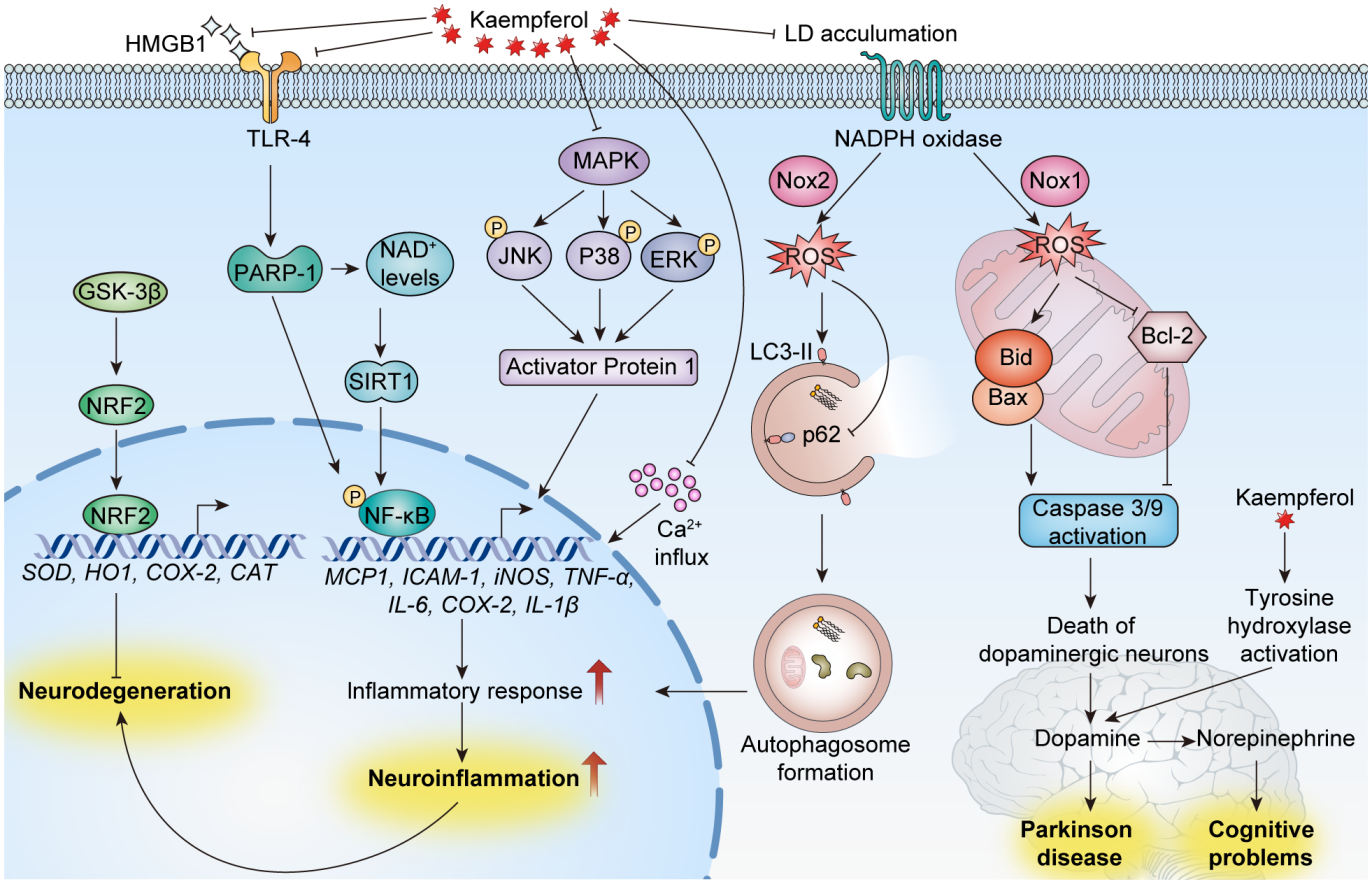
Neuroprotective mechanisms of kaempferol in neurological disorders. Kaempferol, a natural flavonoid, plays a significant role in the prevention and treatment of neurological diseases. It upregulates Nrf2 expression, thereby enhancing cellular antioxidant defenses and suppressing oxidative stress. Simultaneously, it inhibits key pro-inflammatory signaling pathways such as NF-κB and JAK-STAT, alleviating neuroinflammation. Through these coordinated mechanisms, kaempferol demonstrates considerable therapeutic potential in the management of neurodegenerative diseases, including Alzheimer’s disease and Parkinson’s disease.

Research indicates that the natural flavonoid kaempferol has multifaceted regulatory effects on autophagy. In hepatotoxicology, kaempferol treatment significantly reduced liver toxicity, an effect linked to stimulating autophagy, activating AMP-activated protein kinase, and inhibiting oxidative stress (209). In oncology, kaempferol promoted autophagic death in non-small cell lung cancer cells via the PI3K/AKT/mTOR pathway, inhibiting tumor progression (210); similarly, in cervical cancer, it induced tumor cell apoptosis and autophagy via the PI3K/AKT/mTOR pathway, exerting anti-cancer effects (122). Docetaxel combined with kaempferol synergistically induced autophagic death in prostate cancer cells, showing significant anti-cancer synergy (211). In breast cancer research, kaempferol promoted excessive ROS production, inducing autophagy-mediated cell death under low glucose conditions by upregulating LC3-II and p62 expression, inhibiting tumor progression (193). In oral cancer, kaempferol increased p-JNK and phosphorylated p38 kinase expression, promoting JNK-mediated autophagy and inducing tumor cell death (99). In nervous system tumors, kaempferol exhibited potent anti-glioma activity by inducing ROS production, subsequently triggering autophagy and pyroptosis, effectively inhibiting *in vivo* tumor growth (212). In metabolic disease, kaempferol treatment significantly alleviated diabetes-induced albuminuria and glycolipid metabolic dysfunction, mitigating pathological changes like mesangial matrix expansion, glomerular basement membrane thickening, and podocyte loss, potentially by reducing apoptosis and enhancing podocyte autophagy, regulating the AMPK/mTOR pathway (213). In neurodegenerative disease research, kaempferol reduced neuronal death and prevented dopaminergic neurodegeneration in PD by promoting autophagy to alleviate mitochondrial damage from lipid droplet deposition and peroxidation, offering new strategies for treating neurodegenerative diseases (214). However, under certain pathological conditions, kaempferol exhibits inhibitory effects on autophagy. In a lead-induced neurotoxicity model, kaempferol improved cognitive dysfunction and neuronal damage by increasing NMDAR2B and PSD95 levels and inhibiting excessive autophagy (215). In an ischemic stroke model, kaempferol treatment activated protective autophagy via the AMPK signaling pathway, mitigating cerebral I/R injury and exerting neuroprotection (216). Interestingly, in respiratory disease research, kaempferol showed dose-dependent effects in TGF-β1-induced human bronchial epithelial cells, inhibiting NOX4-mediated autophagy by reducing NOX4 levels, improving airway inflammation and remodeling, and playing an important role in treating allergic asthma (217). Based on this evidence, it can be concluded that kaempferol plays a significant molecular regulatory role in the pathology of neuroinflammation by specifically modulating cellular autophagy pathways. This regulation is context-dependent and disease-specific, suggesting kaempferol may function as an autophagy modulator, exerting therapeutic effects by restoring autophagic homeostasis in different pathological states. Its precise regulatory capacity for autophagy pathways makes it a valuable candidate compound for research into treating neuroinflammation-related diseases (**Figure 4**).

## 5 Prospects for the clinical translation of kaempferol

### 5.1 The role of kaempferol in neurodegenerative diseases

Neurological diseases, including neurodegenerative diseases (e.g., AD, PD), stroke, MS, and depression, are among the leading causes of disability and mortality worldwide, placing a heavy burden on healthcare systems (218). Their pathological mechanisms are extremely complex, typically involving interactions among multiple molecular pathways such as imbalance between oxidative stress and antioxidant defense systems, chronic neuroinflammatory responses, mitochondrial energy metabolism dysfunction, disruption of proteostasis leading to misfolding and abnormal aggregation, and aberrant activation of programmed cell death pathways (219–221). Currently, pharmacological treatments for many neurological diseases are primarily limited to alleviating clinical symptoms rather than targeting core mechanisms to halt or reverse pathological progression. Long-term medication often accompanies significant side effects, including cognitive impairment, motor disorders, and metabolic abnormalities (222, 223). Therefore, exploring and developing neuroprotective lead compounds with multi-target regulatory properties from natural product resources, which exert therapeutic effects by synergistically modulating multiple key nodes in the disease network, has become an important strategic direction in neuroscience research. Kaempferol (3,4’,5,7-tetrahydroxyflavone) is a natural flavonoid widely found in various fruits, vegetables, and medicinal plants. Its broad use in daily diet and traditional medicine provides important evidence for its safety profile (224). In recent years, numerous preclinical studies have shown that kaempferol exhibits remarkable pleiotropic neuroprotective potential through its unique chemical structure, capable of simultaneously intervening in multiple key links within the pathological network of neurological diseases. Its mechanisms include, but are not limited to: effectively scavenging ROS and enhancing endogenous antioxidant defense systems; inhibiting aberrant microglial activation and pro-inflammatory cytokine release; modulating the autophagy-lysosome pathway to promote clearance of abnormal protein aggregates; protecting mitochondrial structure and functional integrity; and regulating various cell survival signaling pathways. These multi-target characteristics enable kaempferol not only to alleviate common pathological processes like neuroinflammation and oxidative stress but also to directly intervene in disease-specific proteotoxic aspects, thereby showing unique advantages for early intervention and disease course management in neurodegenerative diseases, offering a highly promising candidate molecule for developing next-generation neuroprotective therapeutic strategies. Substantial recent preclinical evidence confirms that kaempferol, as a natural flavonoid, exhibits excellent multi-target neuroprotective potential, particularly prominent in models of neurodegenerative diseases like AD and PD. In AD research, kaempferol directly inhibits Aβ aggregation and promotes Aβ clearance via enhancing autophagy-lysosomal pathways. It also reduces tau hyperphosphorylation by modulating GSK-3β activity through the PI3K/AKT signaling axis (225). Kaempferol has been proven to have multiple intervention mechanisms, including antioxidant, anti-inflammatory, anti-cholinesterase, and anti-apoptotic effects. Its actions involve the regulation of several key signaling pathways such as Nrf2/SLC7A11/GPX4, TLR4/NF-κB, IRE1/JNK/CHOP, and MAPKs (226). Compared to normal brains, AD patient brain tissue shows significant oxidative stress features, including elevated lipid peroxidation and protein carbonyl content (227). Kaempferol can directly inhibit Aβ deposition while modulating inflammatory signaling pathways like NF-κB, MAPK, and the NLRP3 inflammasome, reducing the expression of pro-inflammatory factors such as IL-1β, IL-6, IL-18, and TNF-α (228). Antioxidatively, kaempferol effectively inhibits excessive ROS generation, increases SOD and GSH activity, and lowers MDA levels. Simultaneously, it exerts anti-apoptotic effects by regulating the expression of apoptosis-related proteins like Bid and Bcl-2. In a AD transgenic Drosophila model, one month of kaempferol intervention not only reduced oxidative stress markers but also significantly delayed the loss of climbing ability and memory function. This protection is attributed to kaempferol reducing Aβ-42 toxicity to neurons, thereby inhibiting lipid peroxidation, protein oxidation, DNA damage, and cell death (229, 230), further confirming the significance of flavonoids in neurological health (231). Additionally, kaempferol protected PC12 and T47D cells from Aβ-induced toxicity (232) and blocked 4-hydroxynonenal-induced PC12 cell apoptosis by directly inhibiting NADPH oxidase (233). In PD research, kaempferol inhibits α-synuclein oligomerization and prevents Lewy body formation. It protects dopaminergic neurons by promoting autophagy-mediated lysosomal degradation of lipid droplets, thereby reducing lipid peroxidation and mitochondrial damage in the substantia nigra (189). Kaempferol delayed the loss of climbing ability and motor function in a dose-dependent manner in PD transgenic flies, reduced oxidative stress damage, and upregulated tyrosine hydroxylase expression. Its mechanism may relate to inhibiting α-synuclein aggregation and preventing Lewy body formation (234). Recent evidence shows kaempferol prevented dopaminergic neuron loss and behavioral deficits in MPTP/p-induced PD mouse models, inhibited MPP+-induced lipid droplet accumulation and apoptosis. Its protective effect is achieved by promoting autophagy-mediated lysosomal degradation of lipid droplets, thereby reducing lipid deposition and peroxidation, alleviating mitochondrial damage, and decreasing lipid oxidative stress in the substantia nigra pars compacta (214). Furthermore, kaempferol can improve motor dysfunction in PD by modulating gut microbiota homeostasis and promoting dopamine release (235). Regarding anti-neuroinflammation, kaempferol shows significant potential, reducing NLRP3 inflammasome formation and the expression of pro-inflammatory factors and pro-apoptotic proteins. By acting on estrogen receptors and inhibiting microglial inflammation, kaempferol plays an important protective role in age-related cognitive impairment (236). In summary, kaempferol demonstrates favorable application prospects in mitigating neurodegenerative disease progression through multi-pathway, multi-target mechanisms of action.

### 5.2 The effect of kaempferol on ischemia-reperfusion injury

I/R injury is a paradoxical phenomenon: tissue damage initially caused by insufficient blood supply (ischemia) is further exacerbated upon restoration of blood flow (reperfusion). This pathophysiological process is critical in various clinical contexts, including myocardial infarction, stroke, and organ transplantation (237–239). The mechanisms of I/R injury are complex and involve multiple factors, including calcium overload, oxidative stress, mitochondrial dysfunction, and a robust inflammatory response, ultimately leading to apoptosis and necrosis. In the search for effective therapeutic strategies to mitigate I/R injury, natural phytochemicals have garnered attention due to their multi-target bioactivities and favorable safety profiles. Kaempferol, a common dietary flavonoid found in vegetables and fruits like broccoli, grapes, and tea, has emerged as a promising candidate. Accumulating preclinical evidence indicates that kaempferol exhibits significant protective potential against I/R injury in multiple organs, including the heart, brain, and kidneys (240). In cerebral I/R models, its neuroprotective effects are closely related to multi-dimensional mechanisms. Studies show kaempferol can effectively inhibit neuronal death and alleviate cerebral I/R injury (159). Specifically, under oxygen-glucose deprivation/reperfusion conditions, neuronal SLC7A11 and GPX4 levels decreased significantly, alongside a weakened endogenous antioxidant system—including NADPH, GSH, and SOD activity. Kaempferol intervention reversed these changes, suggesting protection via the Nrf2/SLC7A11/GPX4 signaling axis, inhibiting the ferroptosis process (159). Beyond anti-ferroptosis, kaempferol also improves long-term neurological function by promoting angiogenesis. Long-term administration was reported to promote vascular neogenesis in the ischemic area by activating the HIF-1α/VEGF-A/Notch1 signaling pathway, thereby improving neurological recovery after ischemic stroke (241). Network pharmacology analysis further supports its multi-target characteristics, suggesting kaempferol may achieve neuroprotection by modulating cellular atrophy and inflammatory responses. Experimental studies confirmed that kaempferol treatment upregulated the BDNF-TrkB-PI3K/AKT signaling pathway, promoted anti-apoptotic protein expression, simultaneously inhibited abnormal glial cell activation, downregulated key inflammatory pathways like COX-2, TLR4/MyD88/NF-κB, and JAK1/STAT3, reduced neutrophil infiltration, and ultimately alleviated neuroinflammation and apoptosis (242). Furthermore, kaempferol plays an important role in BBB stability. BBB permeability increases after cerebral I/R injury, and kaempferol can improve barrier integrity by activating the PI3K/AKT pathway, inhibiting NLRP3 inflammasome activation, and alleviating oxidative stress via the Keap1/Nrf2 pathway; this mechanism was found to be closely related to regulating Lgals3 expression (243).

In conclusion, kaempferol provides a systematic neuroprotective strategy against cerebral I/R injury by synergistically inhibiting neuroinflammation and oxidative stress, and functioning at multiple levels including anti-ferroptosis, promotion of angiogenesis, and maintenance of BBB integrity.

### 5.3 Kaempferol and other neurological diseases

CNS diseases encompass a series of complex disorders including psychiatric diseases, cognitive impairments, and related nervous system tumors. The classic pathophysiological framework of these diseases typically focuses on core aspects such as neurotransmitter imbalance, immune system dysfunction, and oxidative stress damage. Notably, growing clinical evidence indicates the presence of a chronic, low-grade inflammatory state in both the peripheral and CNS of patients with many such disorders (244–247). This neuroinflammation is not a passive accompaniment but a key driver actively involved in their pathogenesis and progression: pro-inflammatory cytokines can directly interfere with the metabolism of key neurotransmitters like dopamine and glutamate, impair synaptic plasticity, inhibit adult neurogenesis, and disrupt the homeostasis of stress response systems like the hypothalamic-pituitary-adrenal axis (248, 249). Against this backdrop, kaempferol, as a multi-target natural compound, is increasingly gaining attention for its therapeutic potential in psychiatric diseases and nervous system tumors. In depression research, kaempferol and its derivatives exhibit significant antidepressant effects. In a corticosterone-induced mouse depression model, a kaempferol derivative directly bound to AMPK, upregulated brain-derived neurotrophic factor expression, and induced autophagy, thereby improving depression-related weight loss and motor deficits, promoting hippocampal neurogenesis, and protecting hippocampal structural integrity (250). In a model of breast cancer-related comorbid depression, kaempferol not only inhibited tumor development but also reduced neuroinflammatory infiltration by modulating the inflammatory balance—specifically, decreasing pro-inflammatory factors IL-1β and IL-6 while increasing anti-inflammatory factors TGF-β1 and IL-10. Simultaneously, it restored levels of monoamine neurotransmitters like serotonin, dopamine, and norepinephrine, exerting antidepressant effects through dual pathways of inflammation and neurotransmitters (9). Furthermore, kaempferol effectively inhibited LPS-induced microglial inflammatory factor secretion, a mechanism involving inhibition of NLRP3 inflammasome activation and modulation of mitophagy, thereby reducing microglial neurotoxicity and ultimately alleviating depression-like behaviors in mice with neuroinflammation (251). In nervous system tumors, kaempferol also demonstrates anti-tumor activity. Studies show it can effectively inhibit neuroblastoma cell proliferation and induce apoptosis. The mechanism involves kaempferol directly binding to the ATP site of inositol-requiring enzyme 1α ribonuclease, thereby inhibiting its activity and hindering the malignant progression and differentiation of neuroblastoma (252). In epilepsy, kaempferol exerts antiepileptic effects by binding to synaptic vesicle glycoprotein 2A, thereby modulating the expression of inflammatory factors such as TNF, IL-6, IL-1β, NF-κB, IL-1Ra, IL-4, and IL-10 (128, 253). In glioblastoma, kaempferol treatment inhibits the proliferation of glioma cells and suppresses tumor growth *in vivo* by activating signaling pathways involving ERK, PKCα, and MMP9, which subsequently induce ROS production and promote autophagy (128, 212). In traumatic brain injury, kaempferol restores cerebral vascular function by modulating Ca²^+^ activation, promoting mitochondrial homeostasis, and improving neurovascular coupling and neural connectivity in brain regions (254).

**Figure 5:**
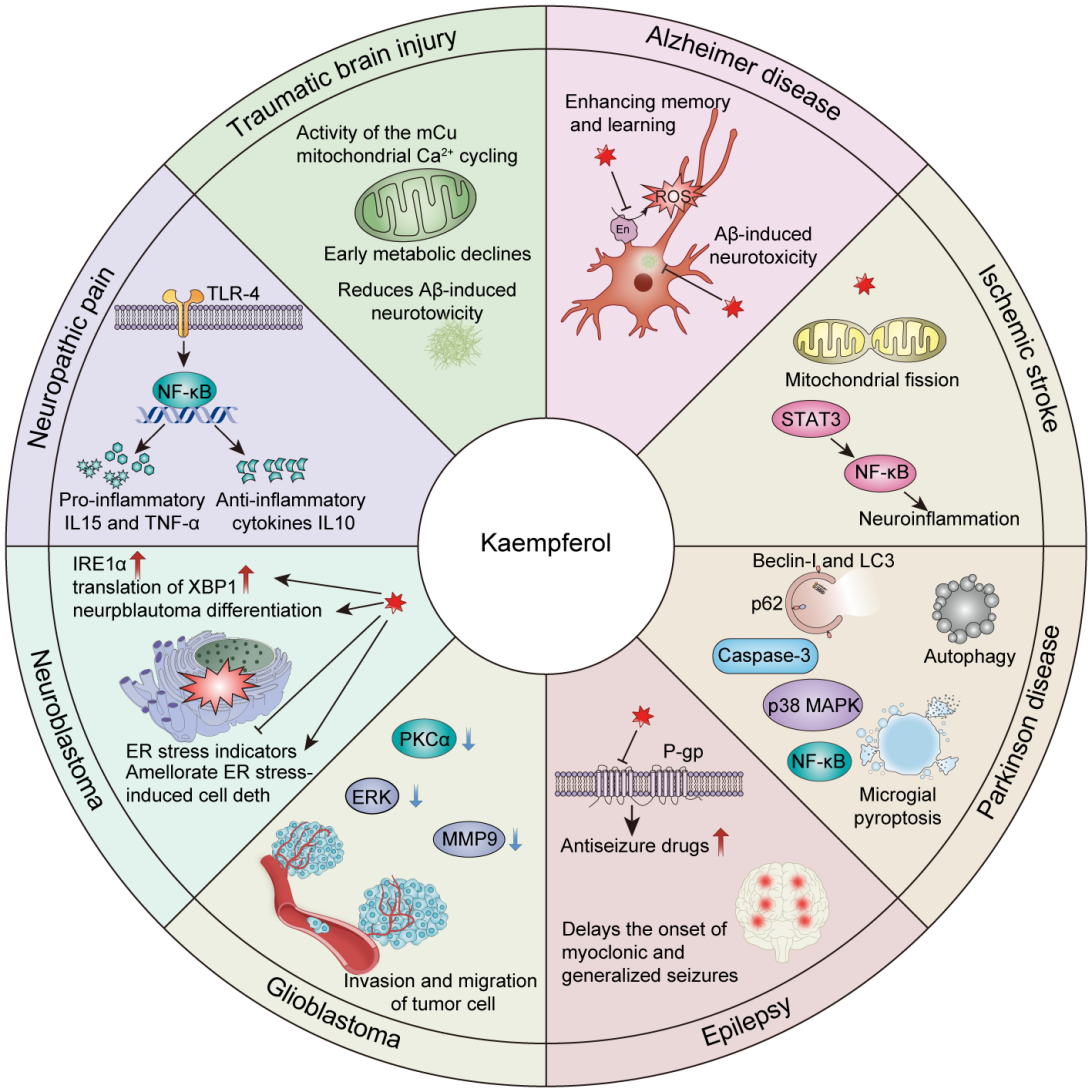
The potential of kaempferol in neuroinflammatory diseases. Neuroinflammation is recognized as a common pathological mechanism underlying various neurological disorders, such as ischemic stroke, Parkinson’s disease, epilepsy, glioblastoma, neuroblastoma, neuropathic pain, and traumatic brain injury. Consequently, kaempferol, functioning as a prodrug, exhibits significant promise for clinical application in these inflammation-associated neurological conditions.

In summary, kaempferol exerts broad protective and therapeutic effects in various CNS disease models through its multi-target properties. Its core mechanism lies in the simultaneous inhibition of the two intertwined pathological processes—neuroinflammation and oxidative stress—and involves precise regulation of specific inflammatory signaling pathways and the endogenous antioxidant system. This pleiotropic characteristic provides a solid theoretical foundation and mechanistic explanation for its further translational research in fields like psychiatric disorders, cognitive impairment, and nervous system tumors (**Figure 5**).

## 6 Conclusion and perspectives

In summary, the existing body of evidence clearly demonstrates that kaempferol, as a multi-target natural bioactive molecule, exhibits significant neuroprotective potential across various neuroinflammation-related diseases. Its mechanisms of action are multifaceted. Primarily, kaempferol directly intervenes in the aggregation of key pathological proteins, such as inhibiting Aβ deposition in AD and α-synuclein oligomerization and Lewy body formation in PD. Secondly, it modulates multiple core signaling pathways, including suppressing the activation of NF-κB and MAPK, and blocking NLRP3 inflammasome assembly, thereby downregulating the production of pro-inflammatory cytokines like IL-1β, IL-6, and TNF-α, and effectively curbing the neuroinflammatory cascade. Furthermore, kaempferol alleviates oxidative stress by enhancing the activities of SOD and GSH, while reducing MDA levels. It also exerts anti-apoptotic effects by modulating the balance of proteins such as Bcl-2/Bid. Notably, its demonstrated ability to promote dopamine release and improve motor function in PD models provides further functional evidence supporting its therapeutic value. Despite these encouraging preclinical findings, the translation of kaempferol into a clinical therapeutic agent faces several challenges. Kaempferol faces significant translational challenges that preclude clinical recommendations despite promising preclinical evidence. Its oral bioavailability is poor (estimated 2% in rodents) due to extensive Phase II metabolism (glucuronidation and sulfation) in the intestine and liver, as well as active efflux by P-glycoprotein (P-gp) and MRP transporters (255). After intravenous administration, kaempferol exhibits rapid clearance (4.40–6.44 L/h/kg) and an extremely short plasma half-life of only 2.93–3.79 minutes (41). Although kaempferol can be detected in brain tissue after systemic administration (peak concentration 0.11 μg/mL), this level is substantially lower than the 8–10 μM concentrations required for neuroprotective effects *in vitro* (256). While kaempferol is generally recognized as safe, comprehensive toxicological data for chronic high-dose therapeutic use are lacking, and no Phase II/III clinical trials have evaluated its efficacy in neurological disorders. Additionally, kaempferol inhibits cytochrome P450 enzymes, particularly CYP3A4 and CYP2C9, and interacts with P-gp, suggesting potential drug-drug interactions with substrates such as warfarin, cyclosporine, and certain dihydropyridines, though clinical relevance remains uninvestigated (257). Collectively, these pharmacokinetic, safety, and efficacy gaps confirm that kaempferol remains a preclinical candidate (255). Current research remains largely confined to cellular and animal models, and its neuroprotective efficacy and pharmacokinetic profile within the complex human system are yet to be fully elucidated. Concurrently, its precise molecular target network requires systematic exploration and validation. A paramount limitation is its inherently low oral bioavailability, which presents a major challenge to its drug development potential. Nanocarrier-based delivery systems: Recent studies have demonstrated that dopamine-coated, kaempferol-loaded metal-organic framework nanoparticles (pDA/KAE@ZIF-8) efficiently penetrate the blood-brain barrier and are significantly taken up by neuronal cells, thereby markedly improving cognitive function (258). Structural modification: Kaempferol-incorporated solid lipid nanoparticles (SLNs), prepared using stearic acid and polysorbate 80 via ultrasonication, can be effectively delivered across the blood-brain barrier to focal cerebral ischemia regions, where they ameliorate damaged neurons and brain structure (259). The application of nanocarriers significantly enhances both bioavailability and drug permeability across the blood-brain barrier. The current evidence does not support clinical recommendations. Therefore, future research efforts should focus on the following key areas: utilizing advanced techniques such as chemical biology to deeply elucidate its molecular targets; developing novel delivery systems to enhance its brain distribution and overall bioavailability; and ultimately, conducting rigorously designed clinical trials to verify its safety and efficacy in humans. Overcoming these hurdles is imperative for kaempferol to evolve from a promising preclinical candidate into a transformative therapeutic agent for the prevention and treatment of neuroinflammation-related diseases.
